# Mesomorphic Behavior of Symmetrical and Unsymmetrical Azomethines with Two Imine Groups

**DOI:** 10.3390/ma2010038

**Published:** 2009-02-06

**Authors:** Agnieszka Iwan, Henryk Janeczek, Bozena Jarzabek, Patrice Rannou

**Affiliations:** 1 Centre of Polymer and Carbon Materials, Polish Academy of Sciences, 34 M. Curie-Sklodowska Street, 41-819 Zabrze, Poland; E-Mails: henryk.janeczek@cmpw-pan.edu.pl (H.J.); bozena.jarzabek@cmpw-pan.edu.pl (B.J.); 2 Laboratoire d’Electronique Moléculaire, Organique et Hybride, UMR5819-SPrAM (CEA-CNRS-Univ. J. FOURIER-Grenoble I), INAC Institut Nanosciences & Cryogénie, CEA-Grenoble, 17 Rue des Martyrs, 38054 Grenoble Cedex 9, France; E-Mail: patrice.rannou@cea.fr (P.R.)

**Keywords:** Symmetrical and unsymmetrical azomethines, Schiff bases, liquid crystals, nematic and smectic phases, polymorphism

## Abstract

Seven symmetrical azomethines with two imine groups (HC=N) were synthesized by condensation of the benzene-1,4-dicarboxaldehyde with five amines (first group: **A1**-**A5**) and of the 2,5-thiophenedicarboxaldehyde with two amines (second group: **AT1**-**AT2**). Additionally, two unsymmetrical azomethines were obtained by a two step condensation of benzene-1,4-dicarboxaldehyde with pyren-1-amine (1^st^ step) (abbreviated hereinafter as **AP1**) and then **AP1** was reacted with 4-dodecylaniline or 4-hexadecylaniline (2^nd^ step) (third group: **AP1A**-**AP1B**). Liquid crystalline properties of the azomethines were studied by differential scanning calorimetry (DSC), polarizing optical microscopy (POM) and UV-vis spectroscopy in the function of temperature [UV-vis(T)]. The Wide-Angle X-ray Diffraction (WAXD) technique was used to probe the structural properties of the azomethines. Mesomorphic behavior was observed for symmetrical and unsymmetrical azomethines, obtained from the benzene-1,4-dicarboxaldehyde and symmetrical ones prepared from 2,5-thiophenedicarboxaldehyde and different amines having aliphatic chains. Based on the POM and DSC measurements the following mesophases were detected: nematic, smectic A, smectic C, smectic F (I), smectic G (J).

## 1. Introduction 

The azomethines, also called Schiff bases, have been widely investigated as liquid crystalline (LC) materials. Special attention has been paid to the study of the LC properties of azomethine oligomers, prompted by their use as building blocks for polymer chains [1]. The liquid crystalline properties of the azomethines could be divided, depending on the number of HC=N bonds in the structure into: (1) Schiff bases having one azomethine (HC=N) bond [2,3,4,5,6,7,8] and (2) symmetrical and unsymmetrical azomethines with two HC=N bonds in the structure [9,10,11,12,13,14,15,16]. Moreover, numerous scientists have investigated the LC properties of symmetrical azomethines with two HC=N bonds in the structure. For example, Ribera *et al*. [9] studied dimeric reactive diglycidyl ethers containing imine mesogens and aliphatic spacers. The authors found that odd-member spacers led to the formation of smectic C mesophases, whereas even-membered spacers led to smectic A and/or nematic mesophases. Ribbera *et al*. also investigated a series of dimeric LC diglycidylester compounds containing imine groups [10]. They found that all compounds exhibited nematic mesophases. Henderson *et al*. [11] characterized a series of semiflexible LC tetramers containing four liquid crystal units (two H_3_CO-Ph-N=N-Ph and two –Ph-HC=N-Ph-) connected via three flexible alkyl spacers. The length of the outer spacers was varied from 3 to 12 units. All the tetramers exhibited an enantiotropic nematic phase. Kishikawa *et al* [12] synthesized one symmetrical azomethine having two imine groups and an OC_3_H_7_ group as outer spacer. The compound exhibited Sm H, Sm G, Sm C, Sm A and nematic phases along with the temperature of isotropisation at about 255 °C. Naito *et al*. [13] found that twin dimers with 3-methyl-pentane spacers behaved as U-shaped molecules forming Sm A phases when the carbon number of the alkyl tail group was longer than 12. On the other hand these authors found that in the case of dimers with linear pentane spacers the antiferroelectric Sm_CA_ was formed [13]. Sudhakar *et al*. [14] investigated the LC properties of the molecules constructed from 1,4-disubstituted benzene rings linked through ester and HC=N units. The outer-spacer was held via n = 2, 4, 6, 8, 10, 12, 14 or 16 aliphatic groups. The lower members of the serie (n = 2-8) were nematogenic, while the higher members (n = 10-14) exhibited both nematic and Sm A phases. The compound with 16 methylene groups presented only Sm A phase. Cozan *et al*. [15] investigated new azomethine sulfone macromers containing benzylideneaniline mesogens. A nematic texture was observed for macromers containing two benzylideneaniline mesogens either connected by etheric (-O-), methylenic links (-CH_2_-) or directly coupled. Mesogenic diols containing one ester group in the center and varying alkoxy spacer length (n = 2, 4, 6, 8, 10) were synthesized by Srinivasan *et al*. [16]. All the diols displayed nematic mesophase. Also a lot of works in the past were dedicated to the liquid crystalline properties of terephthalylidene-bis-4-*n*-alkylanilines [17,18,19,20,21,22,23] with aliphatic chains consisting of five to ten carbon atoms.

The symmetrical azomethines with two HC=N bonds and demonstrated banana-shapes were widely investigated as well [24,25,26,27,28,29,30]. For example, liquid crystalline trimers composed of banana-shaped and rodlike anisometric segments were synthesized and investigated by Yelamaggad *et al*. [24]. The LC azomethines consisted of two ester groups in the center and two cyanobiphenyl groups at the periphery of the compounds. Four alkylene spacers (6, 7, 8, 10) were used as a flexible spacer. All of the trimers displayed an enantiotropic uniaxial nematic phase. Additionally, the compound possessing a heptamethylene (odd-parity) spacer exhibited also a metastable Sm phase.

In this paper we present two groups of symmetrical azomethines and one group of unsymmetrical azomethines with two azomethine bonds. Symmetrical and unsymmetrical azomethines, obtained from benzene-1,4-dicarboxaldehyde, having methyl- or methoxy- aliphatic chains presented polymorphism, while symmetrical azomethines containing carbonitrile- or azo- moiety did not exhibit LC behaviour. Symmetrical azomethines having thiophene ring in the centre also demonstrated mesomorphic behaviour. 

Liquid crystal properties of the azomethines were studied by differential scanning calorimetry (DSC) and polarizing optical microscopy (POM). The structural characterization was performed by NMR and FTIR characteristic completed via X-ray diffraction measurements and optical investigations. The LC behavior as well as photoluminescence properties of the unsymmetrical azomethines were not investigated so far. To best of our knowledge the absorption properties of the azomethines in the function of temperature were first time analyzed hitherto. 

The most remarkable feature of the azomethines that we have investigated was the incorporation of carbonitrile- , azo- pyrene or methyl groups in the symmetrical or unsymmetrical azomethines. It was shown that these groups dramatically influence the thermal and optical properties of the azomethines. Additionally, synthesis and investigation of new mesogens is one of the important and interesting fields for materials research community. 

## 2. Results and Discussion

The azomethines synthesized were new, except for **A2** and **A3**, described in [31,32], respectively, and used here as a references.

### 2.1. Synthesis and characterization

**Figure 1:**
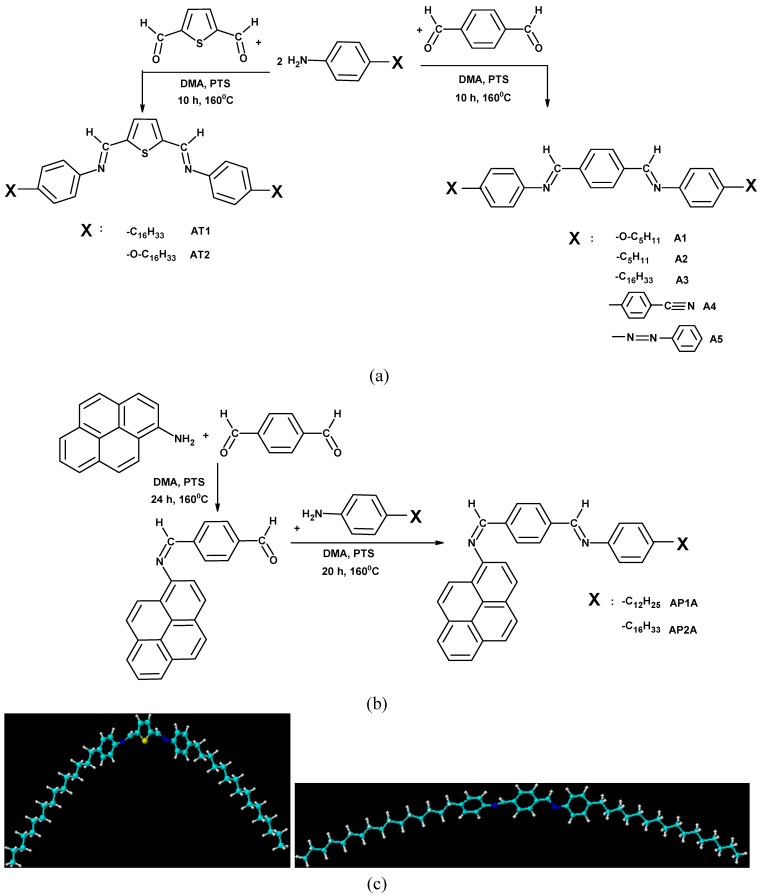
**(**a) Synthetic route to the symmetrical azomethines. (b) Synthetic route to the unsymmetrical azomethines. (c) Geometry optimization of **A3** (right) and **AT1** (left, side view).

The synthesis of the new symmetrical azomethines with two azomethine bonds consisted of one step, while the synthesis route of the unsymmetrical azomethines consisted of two steps. Chemical structures of the symmetrical and unsymmetrical compounds and of the aldehyde are presented in Figure 1, whereas details of synthesis procedures and molecular characteristics along with proton NMR, FTIR, UV-vis, photoluminescence (PL) spectra and elemental analysis of the azomethines and the aldehyde are given in Experimental. The spectral data of all azomethines and aldehyde were found to be consistent with their molecular structure. The absence of the residual amino (NH_2_) and aldehyde (CHO) groups together with the appearance of a typical for azomethine bond (HC=N) band was confirmed by FTIR and NMR spectra. The spectra confirmed both their purity and their molecular structures. 

**Figure 2:**
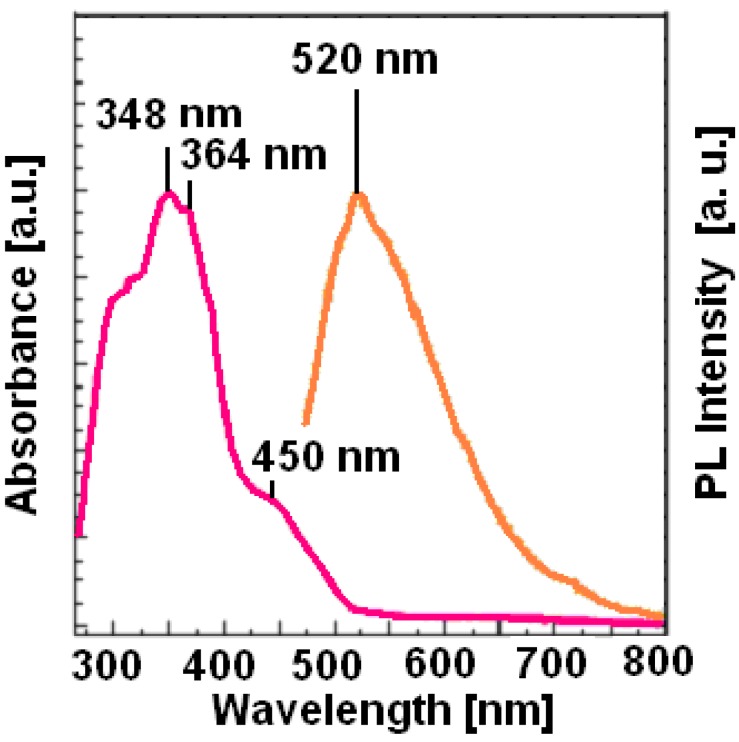
Absorption (UV-vis) and emission (photoluminescence) spectra of the **AP1A** in THF solution (450 nm excitation wavelength).

The UV-vis absorbance spectra of the symmetrical azomethines in solution (CH_3_Cl), except **A5**, displayed two absorption maxima, in the 272-298 nm and 357-362 nm regions (4.56-4.16 eV and 3.47-3.43 eV), whereas the UV-vis spectrum of the **A5** showed one absorption maximum at 384 nm (3.23 eV). In the absorbance spectrum of the unsymmetrical azomethine **AP1A** three main absorption maxima at 318, 348 and about 450 nm were observed (Figure 2). For the same representative samples, the fluorescence (emission) spectra in chloroform solution (1.25 × 10^-3^ M) at room temperature were recorded for the 400 or 450 nm excitation wavelength. For the excitation wavelengths 400 nm, the symmetrical azomethines **A3** and **A4** exhibited one emission band at 535 nm (2.32 eV) and 489 nm (2.54 eV) , respectively, while the unsymmetrical azomethine **AP1A** exhibited one emission band at 520 nm (2.38 eV) under 450 nm excitation wavelengths. 

The Stokes shift for **AP1A** was 70 nm, while for **A3** and **A4** it was 178 and 127 nm, respectively. It is apparent that a hypsochromic effect (blue effect) occurred with the introduction of carbonitrile (**A4**) or pyrene moiety (**AP1A**). These spectroscopic data ascertained the fluorescence properties of these unsymmetrical and symmetrical azomethines. 

As mentioned earlier, several different azomethines have been described in the literature, but only a few azomethines demonstrated fluorescence together with liquid crystalline properties have been reported hitherto [31,32]. 

### 2.2. Mesomorphic behavior

**Figure 3:**
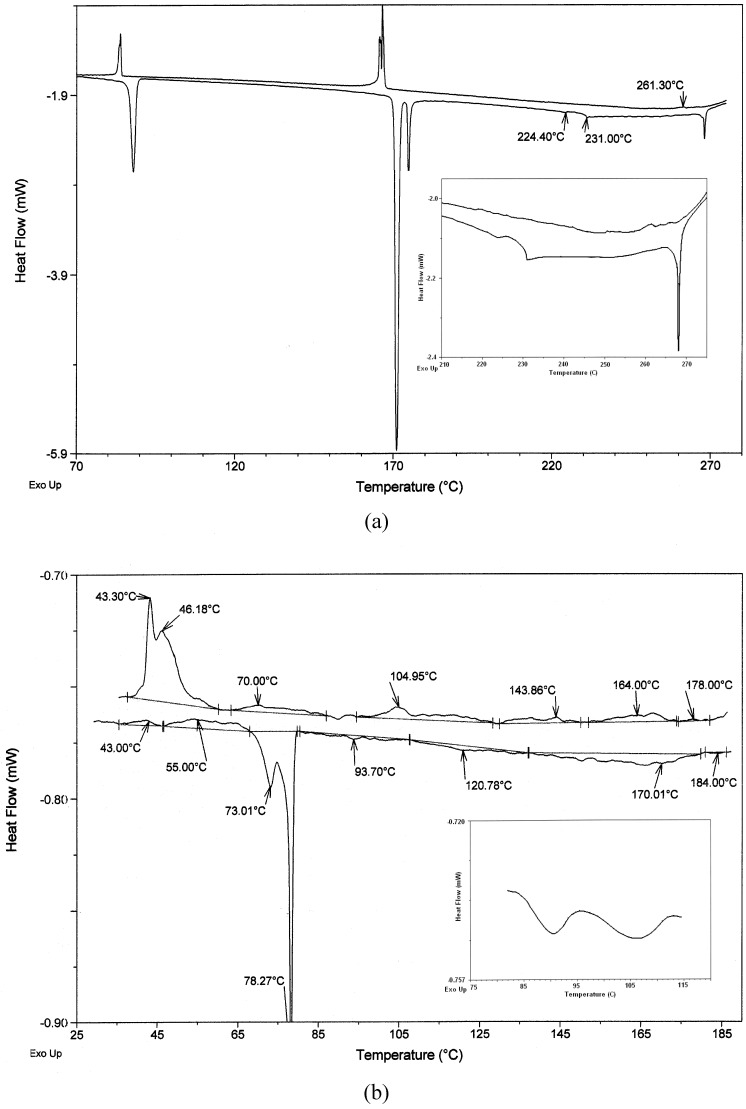
(a) DSC traces of symmetrical azomethine **A1** at a heating/cooling (h/c) rate of 2 °C/min. (b) DSC traces of unsymmetrical azomethine **AP1A** at a h/c rate of 0.8 °C/min (inset heating 2 °C/min from 80 °C to 118 °C).

Liquid crystal properties of these three groups of the azomethines were investigated mainly with the help of differential scanning calorimetry (DSC) and polarizing optical microscope (POM). As examples, DSC thermograms obtained during the first heating and cooling cycles for **A1** and **AP1A** are shown in Figure 3. The tentative mesophases identifications and the scenario (sequence) of phase transitions related to the all compounds are based on the identification of textures appearing in two reference textbooks for liquid crystals [33,34] and on repeated POM and DSC experiments.

The phase sequences, transition temperatures and enthalpies of the symmetrical and unsymmetrical azomethines obtained from benzene-1,4-dicarboxaldehyde are presented in Table 1 and Table 2.

**Table 1:** Identified mesophases and thermal parameters of the azomethines determined by POM.

Phase transition behavior, cooling, detected by POM
**A1**
**I** 277 °C **N** 277 °C till 230 °C **SmC** 220 °C till 172 °C **Cr** < 172 °C
**A2**
**I** 237 °C **N** 237 °C till 216 °C **SmC** 216 °C till 155 °C **Cr**< 160 °C
**A3**
**I** 165 °C **N** 165 °C till 142 °C **SmA** 142 °C till 102 °C **Cr** < 102 °C
**AP1A**
**I** 185 °C **SmA** 177 °C till 183 °C **SmC** 177 °C till 157 °C **SmF (or I)** 157 °C till 127 °C **SmG (or J)** 127 °C till 50 °C **Cr** < 50 °C

I-isotropic, Cr- crystalline, Sm- smectic, N- nematic

#### 2.2.1. Mesomorphic behavior of the symmetrical azomethines **A1**-**A5**

Upon DSC analysis the symmetrical azomethines **A1**-**A3** exhibited a typical transition behavior. Two enantiotropic transitions, crystal-to-mesophase (Cr/M) and mesophase-to-isotropic (M/I), were observed. The clearing temperature decreased with increase the length of the side chains, i.e., 261.3 °C (**A1**) > 230.5 °C (**A2**) > 160.7 °C (**A3**) on cooling cycle (Table 2). Introduction of oxygen atom into **A1** caused increase of melting point and isotropic state of **A1** in comparison with **A2**. The temperature range of the mesophases was decreased with increase the length of side chains (compare **A2** and **A3** in Table 2), as well. Compound A1 showed a crystal to isotropic liquid transition at 268.0 °C with a ΔH=2.9 J/g on the first heating cycle. On cooling from the isotropic state to room temperature, a small exothermic peak appeared at 261.3 °C with a ΔH= 1.7 J/g, corresponding to the transition from the isotropic liquid to the liquid crystalline phase. Compounds **A2** and **A3** have isotropisation at lover temperature (at 230.8 °C and at 160.9 °C) then A1 (Table 2).

The DSC analysis of the compounds **A1**-**A3** showed three, four or six endothermic clearing transitions on the first heating cycle. When cooled from the isotropic liquid state to room temperature, the compounds exhibited three or five exotherms corresponding to the transition from the isotropic liquid to crystalline phase (see Figure 3a). Symmetrical azomethines **A4**-**A5** did not exhibit liquid crystal behavior. 

**Figure 4:**
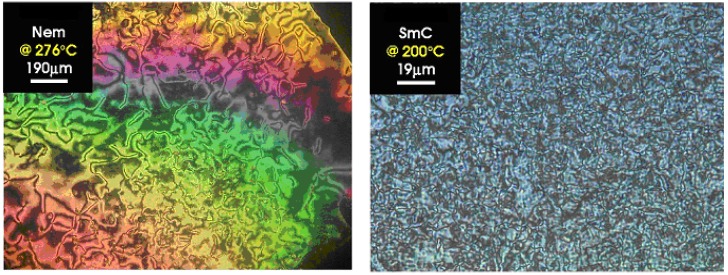
Photomicrographs of the optical textures of mesophases obtained for the symmetrical **A1** (seen at 276 °C and 200 °C).

In the POM and DSC study, the compounds **A1** and **A2** shown stable enantiotropic smectic C (SmC) and nematic (N) liquid crystal phases, while the compound **A3** presented stable enantiotropic smectic A (SmA) and nematic liquid crystal phases (see Table 1). As a representative case, the microphotographs of the nematic and smectic phases obtained for compound **A1** are shown in Figure 4.

**Table 2:** Transition temperatures and enthalpies of the symmetrical and unsymmetrical azomethines detected by DSC.

Phase sequences		
Code	Phase transitions [°C] (corresponding enthalpy changes) [J/g]	
	heating	cooling
**A1**	268.0 (2.9), 231.0 (3.5), 224.4 (0.6), 174.7 (7.8), 171.1 (61.6) 87.9 (20.3)	261.3 (1.7), 166.5 (50.1), 83.9 (16.4)
**A2**	230.8 (3.4), 210.0 (1.9), 147.2 (8.7), 69.0 (42.0)	230.5 (2.6), 209.8 (1.8), 146.5 (8.6), 60.7 (2.9), 47.5 (36.0)
**A3**	160.9 (18.7), 139.0 (14.5), 88.4 (129.1)	160.7 (17.7), 138.4 (13.5), 83.2 (126.1)
AP1A*	182.0 (0.3), 171.0 (2.2), 158.1 (1.8), 131.4 (1.1), 97.1 (1.4), 85.0 (0.9), 78.1 (17.8), 72.8 (38.4), 51.1 (7.4)	181.5 (0.2), 168.3 (6.9), 120.9 (2.1), 94.8 (2.7), 45.6, 43.1 (34.3)
**AP1B**	292.0 (5.2), 171.8 (9.8), 119.0 (4.4), 99.8 (19.5)	275.1 (34.1), 61.6 (6.7)

Peak temperatures in the DSC thermograms obtained during the first heating and cooling cycles. * at a heating/cooling rate of 1 °C/min.

The nematic phase of the symmetrical azomethines **A1**-**A3** and SmC phase of **A1** (see Figure 4) demonstrated Schlieren texture, while smectic C phase of the **A2** exhibits sanded texture (data not presented here) [31,32]. 

**Figure 5:**
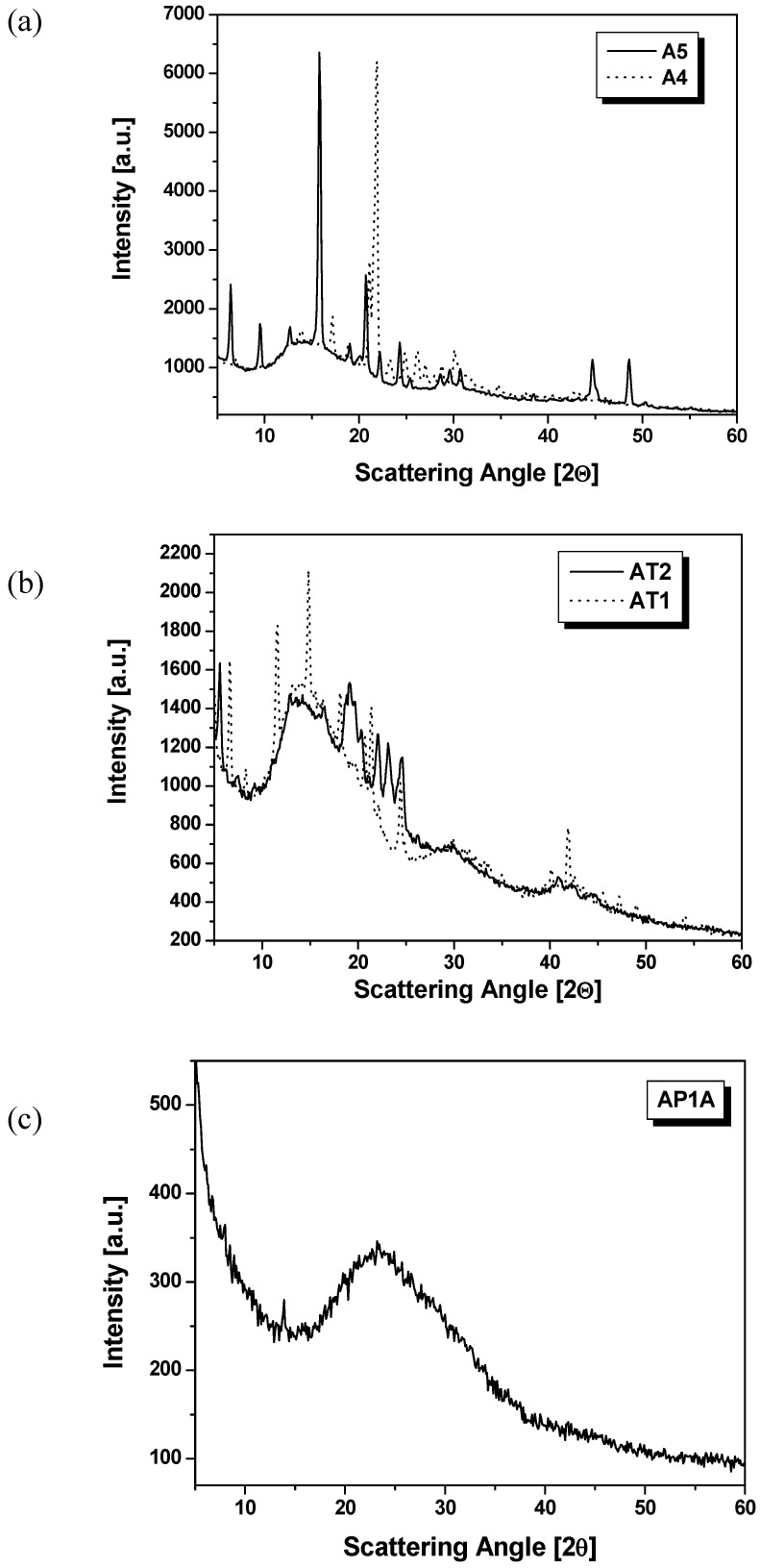
(a) X-ray profiles of symmetrical azomethines **A4** and **A5**. (b) X-ray profiles of symmetrical azomethines **AT1**-**AT2**. (c) X-ray profile of unsymmetrical azomethine **AP1A**. (seen at room temperature).

X-ray diffraction studies of the symmetrical azomethines **A1**-**A5** powders revealed their crystalline nature. The WAXD patterns for the compounds showed very sharp diffraction peaks with several weak diffractions in smaller angles (2Θ scanning), indicating that a highly ordered crystalline structure exists in the azomethines. The wide-angle X-ray diffraction patterns of the **A4** and **A5** over the 2θ range of 5° – 60°, as example, are shown in Figure 5a. 

The reflection peaks (2θ**)** and d spacing from WAXS diffraction diagram of the azomethines **A1**-**A5** are collected in Table 3.

**Table 3:** WAXS data for the azomethines **A1**-**A5**.

Code	WAXS data
	2θ [°] (corresponding d spacing) [Å]
**A1**	**6.45** (13.69), 9.56 (9.24), 15.84 (5.59), 20.00 (4.43), 21.23 (4.18), 22.13 (4.01), 22.89 (3.88), 24.32 (3.66), 25.39 (3.50), 28.73 (3.10), 35.08 (2.56), 38.50 (2.34), 42.80 (2.11), 45.84 (1.98), 48.63 (1.87)
**A2**	5.57 (15.84), 11.31 (7.81), 13.53 (6.54), 17.41 (5.09), **20.56** (4.31), 22.59 (3.93), 24.46 (3.63), 28.91 (3.08)
**A4**	13.80 (6.41), 17.21 (5.15), 18.87 (4.70), 20.98 (4.23), **21.82** (4.07), 23.26 (3.82), 24.86 (3.58), 26.21 (3.40), 26.98 (3.30), 28.04 (3.18), 28.65 (3.11), 30.15 (2.96)
**A5**	6.37 (13.86), 9.56 (9.24), 12.66 (6.98), **15.78** (5.61), 19.03 (4.66), 20.62 (4.30), 22.13 (4.01), 24.24 (3.67), 25.39 (3.50), 28.65 (3.11), 29.55 (3.02), 30.76 (2.90), 44.71 (2.02), 48.57 (1.87)

Bold data indicate the main peak

The symmetrical compounds **A1** and **A2** exhibited one main reflection at 2θ = 6.45° and 20.56°, respectively (d spacing 13.69 Å and 4.31 Å). In the X-ray diffraction pattern of the azomethines **A4** and **A5** one main reflection at 2θ = 21.82° (d spacing 4.07 Å) and 2θ = 15.78° (d spacing 5.61 Å), respectively, were found (Figure 4a). On the other hand, compound **A3** displayed five main reflections at 2θ = 19.55°, 20.56°, 21.15°, 21.80° and 23.45° (d spacing 4.54, 4.31, 4.20, 4.07 and 3.79 Å). The small and wide X-ray diffraction characteristics of the **A3** are presented in [32]. 

#### *2.2.2. Thermal behavior of the symmetrical azomethines* *AT1-AT2*

The symmetrical azomethines **AT1** and **AT2** were synthesized by replaced phenyl ring via thiophene one (Fig 1a). The thermal behavior of these compounds was investigated via DSC and POM techniques. Compounds were investigated by DSC during different heating and cooling cycles. The results of the phase transition (heating scan) are presented below:

**AT1** (heating 0.01 °C/min): CrI 133.3 °C [9.4], CrII 134.4 °C [40.8], SmX 135.5 °C [3.4], I
**AT1** (heating 0.35 °C/min): CrI 103.8 °C [32.9], CrII 134.5 °C [89.3] I
**AT2** (heating 1 °C/min): CrI 99.5 °C [11.8], CrII 129.8 °C [5.8], CrIII 153.5 °C [83.5] SmX1 154.6 °C [10.3], I
**AT2** (heating 0.4 °C/min): CrI 96.4 °C [9.5], CrII 126.2 °C [1.8], CrIII 153.4 °C [87.8], I

**Figure 6:**
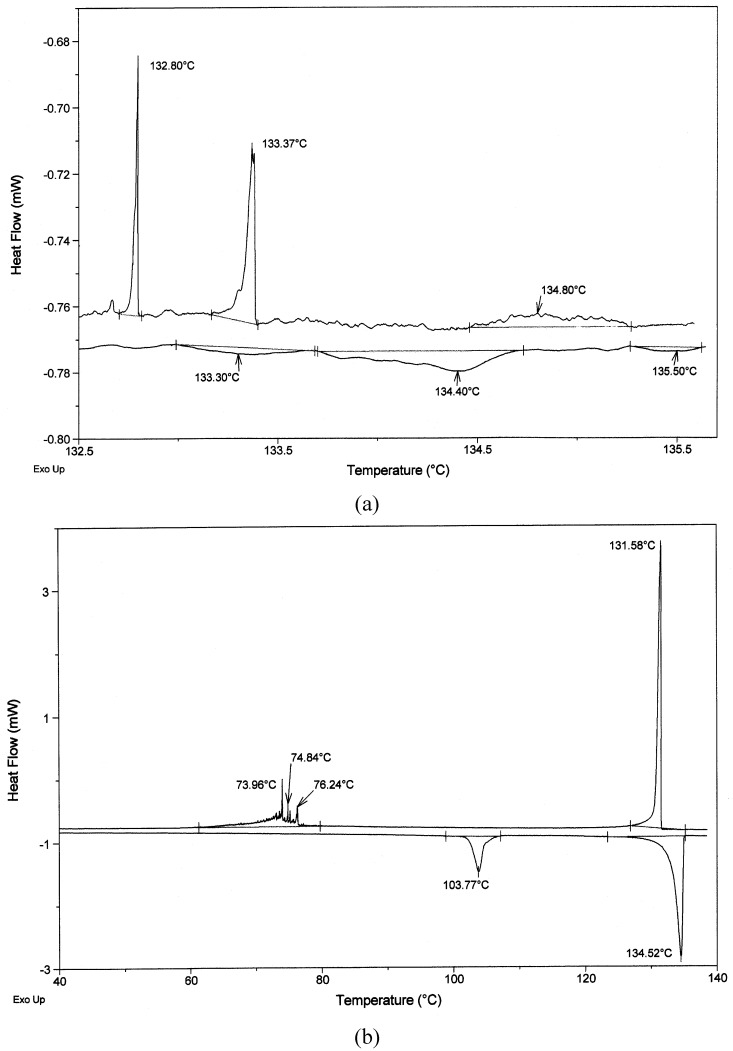

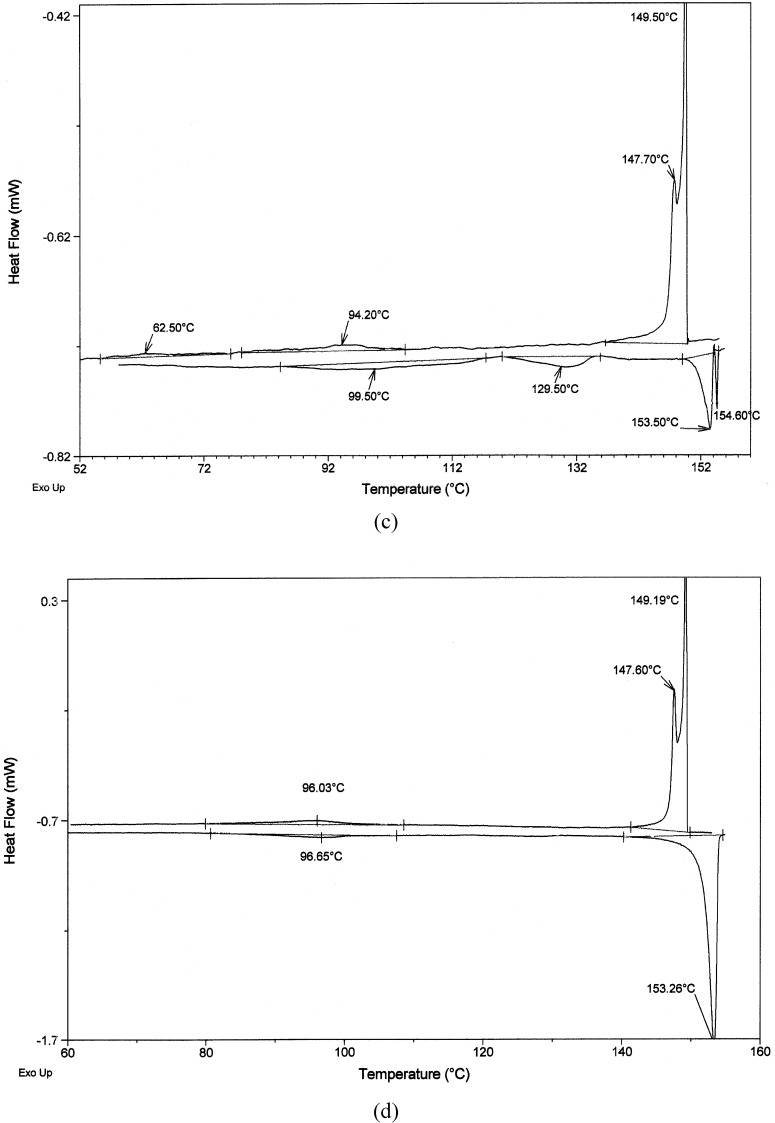

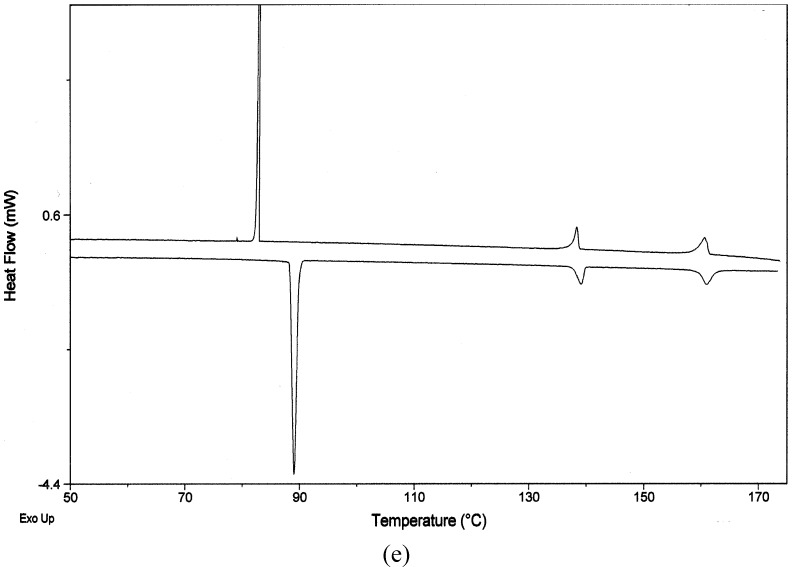
(a) The DSC traces of symmetrical azomethine **AT1** at a h/c rate of 0.01 °C/min and (b) The DSC traces of symmetrical azomethine **AT1** at a h/c rate of 0.35 °C/min. (c) DSC traces of symmetrical azomethine **AT2** at a h/c rate of 1 °C/min and (d) DSC traces of symmetrical azomethine **AT2** at a h/c rate of 0.4 °C/min. (e) DSC traces of symmetrical azomethine **A3** at a h/c rate of 1 °C/min.

The numbers in square brackets under the transition temperatures designate the transition enthalpies in kJ/mol. The symbol Sm X is used here for an unidentified order smectic phase. DSC curves of the compounds **AT1** and **AT2** during different heating and cooling cycles are presented in Figure 6 a-d. Also presented in Figure 6 are the DSC curves of **A3**, for comparison of the thermal behaviors of **AT1** and **A3**.

**Figure 7:**
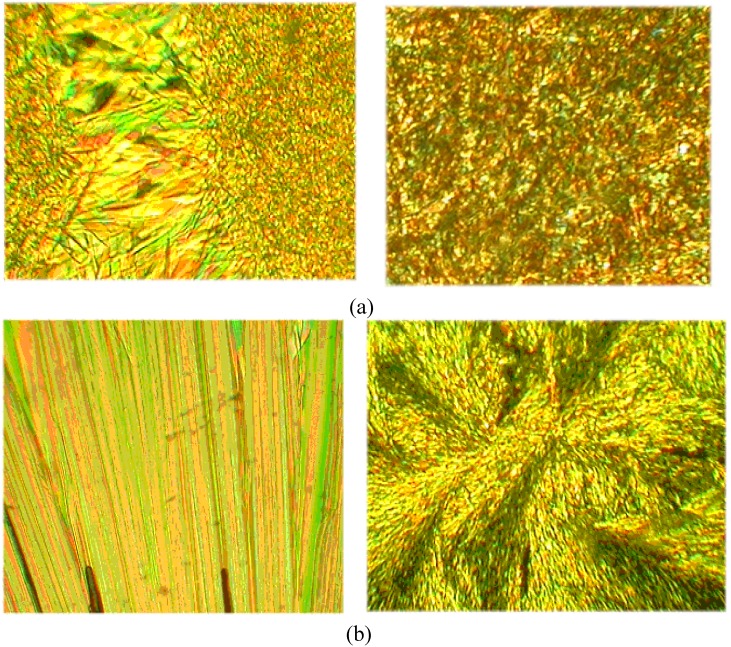
(a) Photomicrographs of the optical textures of mesophases obtained for the symmetrical **AT1** (left, seen at 136 °C) and **AT2** (right, seen at 154 °C). (b) Photomicrographs of the optical textures of crystals obtained for the symmetrical **AT1** (left, seen at 133 °C) and **AT2** (right, seen at 153 °C).

On cooling the isotropic liquid of **AT1** and **AT2** the mesophase appeared as a mosaic texture under the microscope characteristic for the Sm X phase. Moreover, a kind of batonnets texture could be obtained for **AT1** (Figure 7a, left side). Additionally, nice crystals under POM investigations were observed for both samples (Figure 7b). In contrary to the **AT1** optical micrographs of the **AT2** evidence showed “spherulite-like” domains (Figure 7b, right side). 

To account for differences between the thermal behavior of **A3** and **AT1** a preliminary geometry optimalization (ACD/ChemSketch, 3D Structure Optimization) of these compounds was carried out. The average C-C bond length between the C=N linkage and phenylene ring in **A3** was 1.502 Å, while that between the C=N linkage and thiophene ring in **AT1** was 1.545 Å. The N-C bond length in **A3** was 1.512 Å, while in the case of **AT1** it was 1.552 Å. The C=N bond lengths of **A3** and **AT1** were 1.312 Å and 1.319 Å, respectively. Hence, bond length alternation between C-C and C=N was larger in **AT1** (0.226 Å) than that in **A3** (0.19 Å). As shown in Figure 1c, the valence angles between the C=N linkage and the adjacent phenylene ring in **A3** and thiophene in **AT1** were 122.55° and 124.75°, respectively. Also, a significant difference was shown on the valence angle between the C=N linkage and the adjacent N-phenylene ring. **AT1** had a larger valence angle (125.45°), as compared with that (122.40°) of **A3** by 3.05°, which suggests that **A3** had a more planar conformation than **AT1** (see Figure 1c). 

The wide-angle X-ray diffraction patterns of the compounds **AT1** and **AT2** over the 2θ range of 5° – 60° are shown in Figure 5b. The diffraction arising from the crystallites is observed which confirmed crystalline pattern of both compounds. The WAXD patterns of the compounds **AT1**-**AT2** showed very sharp diffraction peaks with several weak diffractions in smaller angles (2Θ scanning), indicating that a highly ordered crystalline structure exists in the azomethines, especially in the **AT1**. The compound **AT1** exhibited three main reflections at 2θ = 5.47°, 12.99° and 16.50° (d spacing 16.14, 6.81 and 5.37 Å, respectively). On the other hand the diffractions arising from the crystallites were observed for **AT2** at 6.61°, 8.31°, 11.50°, 14.78°, 18.07°, 20.66°, 21.42°, 22.09°, 24.46°, 29.84°, 40.19°, 41.89°, 42.62°, 45.50°, 47.27°, 49.03° and 53.90° and demonstrated less sharp diffraction peaks in the range 5-60° in comparison with the **AT1** (Figure 5b). 

Bent-shaped molecules (so-called banana molecules) have attracted great attention in the field of innovative liquid crystal research. Their unique properties of spontaneous chiral symmetry breaking and decoupling of polar and tilt order have been extensively studied for the last decade [24,25,26,27,28,29,30]. There are three main groups of bent-core liquid crystals: (1) molecules incorporating a rather rigid bent aromatic core (i.e. resorcinol derived compounds), (2) mesogenic dimers with an odd-number flexible spacer unit and (3) hockey-stick molecules, where an alkyl chain is attached to the meta-position at one end of an aromatic core [30]. In this paper two bent-shaped azomethines **AT1** and **AT2** were synthesized and investigated via DSC, POM and WAXD techniques. In X-ray diffraction measurements, a lot of sharp diffraction peaks were observed at wide angles and additionally several sharp peaks at small angles, as shown in Figure 5b. From these results the azomethines **AT1** and **AT2** probably exhibited B3 phase. This part of our work needs more investigations. 

#### *2.2.3. Mesomorphic behavior of the unsymmetrical azomethines* **AP1A***-***AP1B**

**Figure 8:**
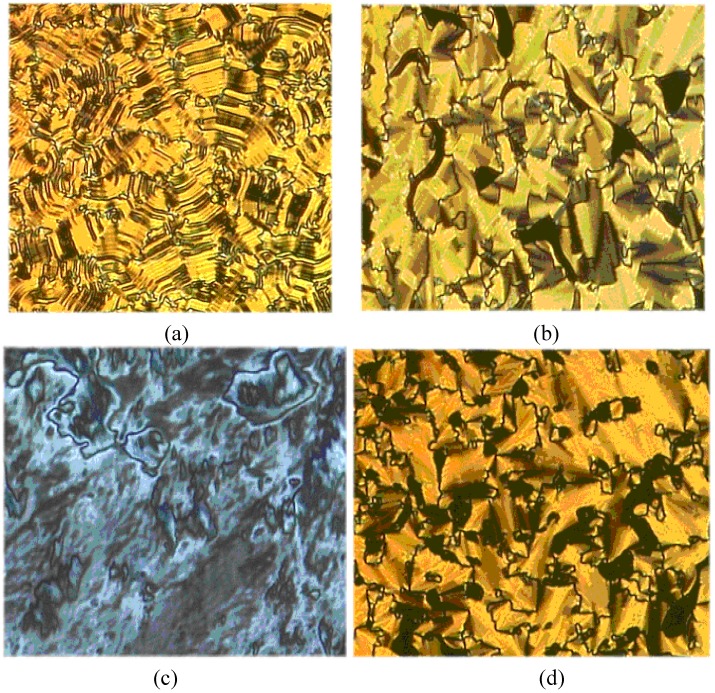
Photomicrographs of the optical textures of mesophases obtained for the unsymmetrical azomethine **AP1A**. The banded focal-conic fan texture of the smectic G (or J) phase (seen at 120 °C, a), the broken focal-conic texture of the SmF (or I) phase (seen at 135 °C, b), the schlieren texture of the SmC phase (seen at 157 °C, c) and the focal-conic fan texture of the SmA phase (seen at 170 °C, d).

All phase transition parameters of the unsymmetrical **AP1A** were determined by the DSC and POM technique and are presented in Table 1 and Table 2. Typical DSC traces of **AP1A** are shown in Figure 3b. This unsymmetrical azomethine exhibited very rich smectic polymorphism, as is shown in Table 1 and Table 2 and in Figure 3b. The POM and DSC observations revealed that **AP1A** is an enantiotropic liquid crystal exhibiting four phases (SmG, SmF, SmC, SmA). In contrast, the unsymmetrical **AP1B** exhibited two enantiotropic smectic phases, which implies a significant effect of the length of the aliphatic chain on the mesomorphic properties. The identification of the smectic phases was confirmed by POM. Microphotographs of the **AP1A** are presented in Figure 8.

Four kinds of textures were observed for **AP1A**: (1) the banded focal-conic fan texture of the smectic G (or J) phase (Figure 8a at 120 °C), (2) the broken focal-conic texture of the Sm F (or I) phase (Figure 8b at 135 °C), (3) the schlieren texture of the SmC phase (Figure 8c at 157 °C) and (4) the focal-conic fan texture of the SmA phase (Figure 8d at 170 °C).

It is very difficult to find changes between SmF and SmI or SmG and SmJ. The only change which occurs at the transition from SmF to SmI is one of tilt direction (SmF: tilt direction towards an edge of the hexagon while SmI: tilt direction towards an apex of the hexagon) [34]. Both SmF and SmI are tilted biaxial (2D) smectic mesophase in which the mesogenic molecules are hexagonally packed. They both share short range hexagonal order with no long-range correlations in between their smectic layers. 

From a structural organization point of view, a transition from a SmI mesophase to a SmJ one (or from a SmF to a SmG mesophase) would imply the transition from an organization with titled 2D hexagonally packed LC molecules towards the one of titled 3D pseudo-hexagonally packed LC molecules. Put it differently a SmI > SmJ (or a SmF > SmG) transition results in a net gain of positional order of the LC molecules as well as by a transition from a 2D to 3D mesophase. When the SmG phase is formed by cooling a SmF phase the tilt direction with respect to the hexagonal net is retained it means that till towards the edge of the hexagon [34]. 

It is quite difficult to differentiate SmF and SmI mesophases unless they are present in a sequence of mesophases for a specific LC. Nevertheless detailed X-Ray diffraction measurements, neutron scattering studies and POM investigations could be used to highlight such a tiny difference. Additionally, DSC studies could be of great help in the case of LCs compounds show a mesophases SmF and SmI, which have usually enthalpy value in the range of 0.1-0.2 kJ/mol. 

Table 2 reveals that the compound **AP1A** exhibited a clearing point temperature lower than **AP1B**. On the other hand the compound **AP1B** had the highest clearing point temperature in comparison with another azomethines (Table 2). Also the melting temperature of **AP1B** was the highest in comparison with the other azomethines. The following order was observed: 69 °C (**A2**) < 72.8 °C (**AP1A**) < 87.9 °C (**A1**) < 88.4 °C (**A3**) < 99.8 °C (**AP1B**). 

**Figure 9:**
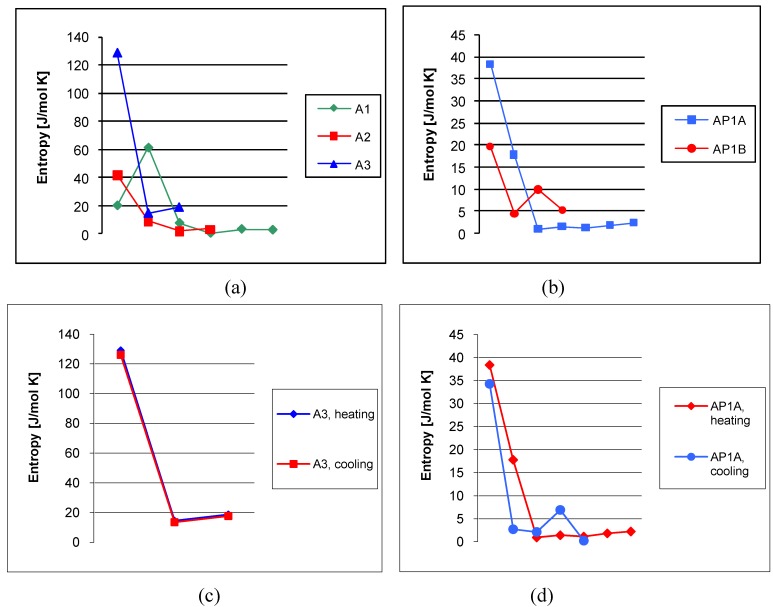
(a-b) Influence of the amine structure on the effect of entropy of the azomethines **A1**-**A3** and **AP1A**-**AP1B** during heating process. (c- d) Influence of the heating and cooling process on the effect of entropy of the azomethines **A3** and **AP1A**.

Additionally, the phase transitions were analysed based on their entropy values (Figure 9). The melting process has the highest entropy values. The entropy values decreased from 129.1 J/mol K for A3 to the minimal values 19.5 and 20.3 J/mol K for **AP1B** and **A1**, respectively. The parameters of the heating and cooling process were more or less similar (Figure 9c-d). 

The X-ray diffraction pattern obtained from **AP1A** is presented in Figure 5c. A broad halo and a sharp reflection at wide angles are shown in Figure 5c. One broad diffraction peak of diffusion type centered at 23.33° (2θ) and one sharp reflection at 13.82° was observed for **AP1A**. 

### 2.3. UV-vis(T) Studies

**Figure 10:**
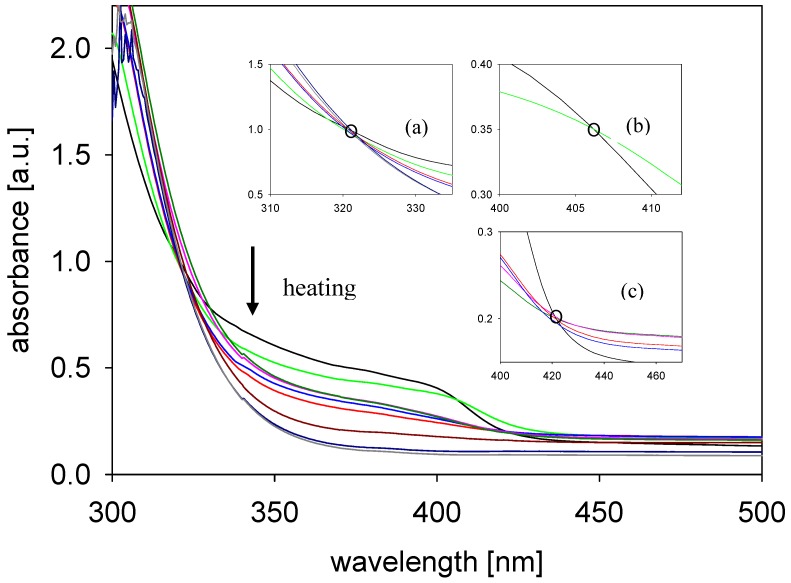
Temperature dependence of UV-vis spectra of the **A2** at the following temperatures: 25, 70, 150, 155, 185; 200, 215, 230, 237 °C. Insets: (a) The isosbestic point at 322 nm (for the curves crossed at the following temperatures 25, 70, 150, 155, 230, 237 °C), (b) The isosbestic point at 406 nm (25 and 70 °C) and (c) The isosbestic point at 420 nm (25, 150, 155, 185; 200 °C).

The absorption properties of the azomethines in the function of temperature were first time analyzed hitherto. Figure 10 shows the temperature dependence of the UV-vis spectra of **A2**, as an example, in a temperature range from room temperature to the clearing point in the heating process.

Using UV-vis spectroscopy to monitor the changes in spectroscopic properties of **A2** during heating, three changes in the position of the isosbestic point were observed, indicating that the differential transitions proceeds not randomly but stepwise. An isosbestic point appears when a compound is quantitatively transformed from crystal to one mesophase and later into another during heating, so the three different isosbestic points observed suggest that three different transitions were successively formed. The spectra of the **A2** gradually changed, with an isosbestic point at 406 nm due to the increase the tempararture from 25 °C to 70 °C (Figure 10, inset b). The isosbestic point at 420 nm appeared for the following temperatures 25, 150, 155, 185, 200 °C, and was about 14 nm red shifts in comparison with the isosbestic point observed at 406 nm (Figure 10, inset c). The third isosbestic point observed at 322 nm (for the temperatures 25, 70, 150, 155, 230, 237 °C, Figure 10, inset a) was about 90 nm blue shifts in comparison with another isosbestic points. 

Also in the UV-vis spectra of **A2** a bathochromic effect with temperature increase was observed. The absorption band around 394 nm at 25 °C was about 10 nm bathochromically shifted, along with increase the temperature to 70 °C (404 nm). At 150 °C the absorption band corresponding to the azomethine bond was broader and not well defined and was observed at this position to 185 °C what correspond to the transition Cr-SmC. Along with increase the temperature from 200 to 237 °C the absorption band disappeared along with decreased the absorption intensity (hypochromic effect).

It should be mentioned that such LC compounds which exhibit a mesophase or a sequence of mesophases over a broad temperature range could be used in: (i) color information technology-based applications [35] for thermally stable glassy derived from cholesterol LC molecules and (ii) organic electronic devices (organic light-emitting devices, organic solar cells). Obtained compounds could be utilized in devices which operating temperature is much higher than room temperature. Moreover, such types of LCs compounds have been used in mixture with other LCs for LC displays. Particularly important are twisted nematic (TN) cells which rely on calamitic mesogens, and usually exhibit high clearing temperatures.

## 3. Experimental Section 

### General

Acetone, ethanol, *N,N*-dimethylacetamide (DMA) (Aldrich) 4-hexadecylaniline (Aldrich), 4-*n*-hexadecyloxyaniline (Alfa Aesar), 4-*n*-dodecylaniline (Aldrich), 4-pentylaniline (Aldrich), 4-pentyl-oxyaniline (Aldrich), 4’-aminobiphenyl-4-carbonitrile (Aldrich), 4-((4-aminophenyl)diazenyl)-*N,N*-dimethylbenzenamine (Aldrich), pyren-1-amine (Aldrich), 2,5-thiophenedicarboxaldehyde and benzene-1,4-dicarboxaldehyde were used without any purification. Synthesized compounds were characterized by ^1^H-NMR and elemental analysis. Compounds were also characterized by Fourier transform infrared (FTIR-ATR) and ultraviolet-visible (UV-vis) absorption spectroscopy. NMR was recorded on a Bruker AC 200 MHz instrument. Chloroform-*d* (CDCl_3_) containing TMS as an internal standard were used as solvent. Elemental analyses (C, H and N) were recorded on a 240C Perkin-Elmer analyzer. FTIR spectra of the compounds were recorded on a Perkin-Elmer paragon 500 spectrometer (wavenumber range: 400-4000 cm^-1^; resolution: 2 cm^-1^). Solution UV-vis absorption spectra were recorded using a Hewlett-Packard 8452A spectrophotometer. X-ray diffraction patterns were recorded using powder samples on a wide-angle HZG-4 diffractometer working in typical Bragg geometry. CuK_α_ radiation was applied.

The phase transitions and mesogenicity were studied by differential scanning calorimetry (DSC) and polarizing microscope observations (POM). DSC were measured on a TA-DSC 2010 apparatus using sealed aluminium pans under nitrogen atmosphere at a heating/cooling rate 0.5 °C/min in a temperature range from –20 °C to over the clearing point. 

The textures of the liquid-crystalline phase were observed with a Polarized Optical Microscopy (POM), set-up composed of: i) LEICA DMLM Microscope (Magnification: 2.5x, 5x, 10x, 20x and 50x) working in both transmission and reflexion modes, ii) LINKAM LTS350 (-196 °C till +350 °C) Hot plate and LINKAM CI94 temperature controller, iii) JVC Numeric 3-CCD KYF75 camera (resolution: 1360 x 1024). 

The temperature dependence of the UV-vis spectra was measured for thin film on the glass (film cast from dichloroethane) by JASCO V-570 UV-Vis-NIR spectrometer using a temperature-controlled optical cell in a temperature range from the room temperature to clearing point in the heating process.

### 3.1. Synthesis of symmetrical azomethines

The symmetrical azomethines were prepared using the synthetic routes shown in Scheme 1a, and identical to those used to prepare the terephthalylidene-bis-4-*n*-alkylanilines [31,32]. Generally, a solution of dialdehyde (1.0 mmol) in DMA (5 mL) was added to a solution of amine (2.0 mmol) in DMA (5 mL) with 0,06 g of *p*-toluenesulfonic acid. The mixture was refluxed with stirring for 10 hours. Then the compound was filtered, washed with ethanol and later with acetone and dried at 60 °C under vacuum for 12 hours.Azomethines A2 and A3 were synthesized and described previously [31,32] and were used as references in this paper. 

*N,N’-(1,4-phenylenebis(methan-1-yl-1-ylidene)bis(4-(pentyloxy)benzenamine)* (**A1**): Yield: 90%; ^1^H- NMR (ppm): δ= 8.54 (s, 2H, 2 × HC=N), 7.98 (4H, s, 4 × H_Ar_), 6.93-7.29 (m, 8H, 8 × H_Ar_), 3.96-4.00 (m, 4H, 2 × OCH_2_), 1.89-1.93 (m, 4H, 2 × CH_2_), 1.41-1.45 (m, 4H, 2 × CH_2_), 0.92-0.97 (m, 6H, 2 × CH_3_); FTIR: 2935, 2859, 1618 (HC=N), 1574, 1500, 1464, 1390, 1359, 1315, 1285, 1236, 1192, 1162, 1109, 1052, 1017, 951, 868, 837, 772, 728, 640 cm^-1^; Anal. Calcd for C_30_H_36_N_2_O_2_ (456.28): C, 78.91%; H, 7.95%; N, 6.13%. Found: C, 79.02%; H, 7.82%; N, 6.20%; Mp: 173 °C; UV-vis in chloroform λ_max_ at 292 and 379 nm; PL in chloroform λ_max_, very low (excitation 400 nm).

*N,N’-(1,4-phenylenebis(methan-1-yl-1-ylidene)bis(4-pentylbenzenamine)* (**A2**): [31]. Yield: 88%; ^1^H- NMR (ppm): δ= 8.52 (s, 2H, 2 × HC=N), 7.98 (4H, s, 4 × H_Ar_), 7.19-7.21 (m, 8H, 8 × H_Ar_), 2.60-2.63 (m, 4H, 2 × CH_2_), 1.32-1.36 (m, 4H, 2 × CH_2_), 1.61-1.64 (m, 4H, 2 × CH_2_), 0.90-0.92 (m, 6H, 2 × CH_3_); FTIR: 2951, 2923, 2854, 1623 (HC=N), 1598, 1562, 1499, 1467, 1419, 1364, 1303, 1192, 1171, 1116, 1013, 970, 884, 852, 839, 812, 728, 591, 566, 551 cm^-1^; Anal. Calcd for C_30_H_36_N_2_ (424.29): C, 84.86%; H, 8.55%; N, 6.60%. Found: C, 84.92%; H, 8.61%; N, 6.80%; Mp: 150 °C; UV-vis in chloroform λ_max_ at 272 and 360 nm; PL in chloroform λ_max_, very low (excitation 400 nm).

*N,N’-(1,4-phenylenebis(methan-1-yl-1-ylidene)bis(4-hexadecylbenzenamine)* (**A3**) [32]. Yield: 81%; ^1^H-NMR (ppm): δ= 8.53 (s, 2H, 2 × CH=N-), 8.00 (s, 4H, 4 × H_Ar_), 7.20-7.24 (m, 8H, 8 × H_Ar_), 2.60-2.66 (m, 4H, 2 × CH_2_-Ar), 1.56-1.63 (m, 4H, 2 × CH_2_-CH_2_-CH_2_-Ar), 1.26 (m, 52H, 2 × (CH_2_)_13_-CH_3_), 0.86-0.90 (m, 6H, 2 × CH_3_); FTIR: 3031, 2954, 2918, 2849, 1622 (HC=N), 1601, 1498, 1472, 1463, 1415, 1357, 1307, 1198, 1168, 1116, 1012, 971, 880, 842, 828, 807, 728, 719, 582, 557, 524 cm^-1^; Anal. Calcd. for C_52_H_80_N_2_ (732.63): C, 85.18%; H, 11.00%; N, 3.82%. Found: C, 85.35%; H, 10.96%; N, 3.95%; Mp: 92 °C; UV-vis in chloroform λ_max_ at 294 and 357 nm; PL in chloroform λ_max_ at 535 nm (excitation 400 nm).

*4’,4”-(1,4-phenylenebis(methan-1-yl-1-ylidene))bis(azan-1-yl-1-ylidene)dibiphenyl-4-carbonitrile* (**A4**) Yield: 75%, ^1^H-NMR (ppm): δ= 8.59 (s, 2H, 2 × CH=N-), 8.06 (s, 4H, 4 × H_Ar_), 7.61-7.74 (m, 4H, 4 × H_Ar_), 7.40-7.39 (m, 4H, 4 × H_Ar_); FTIR: 2224 (C≡N), 1625 (HC=N), 1603, 1594, 1562, 1487,1397, 1364, 1295, 1193, 1175, 1115, 1004, 973, 888, 860, 837, 822, 741, 545 cm^-1^; Anal. Calcd. for C_34_H_22_N_4_ (486.57): C, 83.93%; H, 4.56%; N, 11.51%. Found: C, 83.35%; H, 4.64%; N, 11.41%; Mp: 282 °C; UV-vis in chloroform λ_max_ at 298 and 362 nm; PL in chloroform λ_max_ at 489 nm (excitation 400 nm).

*N,N’-(1,4-phenylenebis(methan-1-yl-1-ylidene))bis(4-(phenyldiazenyl)benzenamine).* (**A5**). Yield: 70%; ^1^H NMR (ppm): δ= 8.60 (s, 2H, 2 × CH=N-), 8.08 (s, 4H, 4 × H_Ar_), 7.93-8.03 (m, 4H, 4 × H_Ar_), 7.38-7.54 (m, 4H, 4 × H_Ar_); FTIR: 2882, 1617 (HC=N), 1582, 1485,1463, 1440, 1414, 1357, 1302, 1287, 1261, 1220, 1186, 1152, 1110, 1103, 1070, 1017, 967, 918, 855, 764, 730, 686, 566, 531, 474 cm^-1^; Anal. Calcd. for C_32_H_24_N_6_ (492.57): C, 78.03%; H, 4.91%; N, 17.06%. Found: C, 78.35%; H, 4.99%; N, 16.95%; Mp: 248 °C; UV-vis in chloroform λ_max_ at 384 nm; PL in chloroform λ_max_ not detected under excitation 400 nm.

*N-(4-hexadecylphenyl)-N-[(1)-(5-{-[(4-ethylphenyl)imino]methyl}thien-2-yl)methylene]amine.* (**AT1**). Yield: 85%; ^1^H-NMR (ppm): δ= 8.63 (s, 2H, 2 × CH=N-), 7.51 (s, 2H, 2 × H_th_), 7.24 (m, 8H, 8 × H_Ar_), 2.62-2.70 (m, 4H, 2 × CH_2_-Ar), 1.60-1.66 (m, 4H, 2 × CH_2_-CH_2_-CH_2_-Ar), 1.30 (m, 52H, 2 × (CH_2_)_13_-CH_3_), 0.89-0.94 (m, 6H, 2 × CH_3_); FTIR: 2912, 2837, 2364, 2328, 1614 (HC=N), 1583, 1500, 1464, 1416, 1364, 1293, 1285, 1241, 1192, 1171, 1114, 1061, 1008, 956, 903, 859, 833, 815, 785, 710 cm^-1^; Anal. Calcd. for C_50_H_78_N_2_S (739.23): C, 81.24%; H, 10.64%; N, 3.79%. Found: C, 81.21%; H, 10.65%; N, 3.67%; Mp: 136 °C; UV-vis in dichloroethane (partially soluble) λ_max_ at 230, 250 shoulder and 385 nm.

*AT2: N-(4-hexadecyloxyphenyl)-N-[(1)-(5-{-[(4-ethoxyphenyl)imino]methyl}thien-2-yl)methylene]am-ine.* Yield: 80%. ^1^H NMR: not soluble in DMSO, not fully soluble in CDCl_3_. FTIR: 2923, 2853, 2356, 2327, 1614 (HC=N), 1587, 1504, 1473, 1460, 1364, 1280, 1245, 1166, 1109, 1021, 951, 938, 837, 728 cm^-1^. Anal. Calcd. for C_50_H_78_N_2_O_2_S (771.23): C, 77.87%; H, 10.19%; N, 3.63%. Found: C, 77.67%; H, 9.99%; N, 3.68%. Mp: 153 °C. UV-vis in dichloroethane (partially soluble) λ_max_ at 230, 250, 296 and 352 nm.

### 3.2. Synthesis of 4-[-(1H-pyren-8-ylimino)methyl]benzaldehyde aldehyde *(**AP1**)*

The aldehyde was prepared using the synthetic routes shown in Scheme 1b. Solution of benzene-1,4-dicarboxaldehyde (1.0 mmol) in DMA (5 mL) was added to a solution of pyren-1-amine (0.5 mmol) in DMA (5 mL) contaning 0.03g of *p*-toluenesulfonic acid. The mixture was refluxed with stirring for 24 hours. The final compound was washed with ethanol and with acetone and dried at 60 °C under vacuum for 12 hours. Yield: 75%. ^1^H-NMR (ppm): δ= 10.19 (s, 1H, HC=O), 8.90 (s, 1H, CH=N-), 8.03-8.35 (m, 9H, H_Ar_, pyrene structure), 7.81-7.90 (m, 4H, 4 × H_Ar_); FTIR: 3042, 2852, 2355, 1688 (HC=O), 1605 (HC=N), 1587, 1478, 1407, 1377, 1346, 1293, 1241, 1197, 1171, 1140, 1092, 1008, 956, 899, 833, 759, 715, 684, 622 cm^-1^; Anal. Calcd. for C_24_H_15_NO (333.38): C, 86.46%; H, 4.54%; N, 4.20%. Found: C, 86.21%; H, 4.65%; N, 4.27%; Mp: 321 °C; UV-vis in dichloroethane λ_max_ at 275, 315, 343, 389 and 435 nm. 

### 3. 3. Synthesis of unsymmetrical azomethines

The unsymmetrical azomethines were prepared using the synthetic route shown in Scheme 1b. Generally, a solution of aldehyde AP1 (1.0 mmol) in DMA (5 mL) was added to a solution of amine (1.0 mmol) in DMA (5 mL) with 0.06 g of *p*-toluenesulfonic acid. The mixture was refluxed with stirring for 20 hours. Then the compound was filtered, washed with ethanol and later with acetone and dried at 60 °C under vacuum for 12 hours.

*N-(4-((4-doadecylphenylimino)methyl)benzylidene)pyren-1-amine.* (**AP1A**). Yield: 70%; ^1^H-NMR (ppm): δ= 8.90 (s, 1H, CH=N-Ar_pyrene_), 8.58 (s, 1H, CH=N-), 7.84-8.04 (m, 9H, H_Ar_, pyrene structure), 7.84-7.89 (m, 4H, 4 × H_Ar_), 7.25-7.30 (m, 4H, 4 × H_Ar_), 2.63-2.71 (m, 2H, CH_2_-Ar), 1.60-1.67 (m, 2H, Ar-CH_2_-CH_2_-), 1.31 (m, 18H, (CH_2_)_9_-CH_3_), 0.93-0.95 (m, 3H, CH_3_); FTIR: 2918, 2842, 2356, 2333, 1622 (HC=N-), 1596, 1561, 1504, 1451, 1412, 1350, 1298, 1197, 1166, 1114, 1096, 1008, 969, 885, 824, 780, 706, 684, 662, 614 cm^-1^; Anal. Calcd. for C_42_H_44_N_2_ (576.81): C, 87.45%; H, 7.69%; N, 4.86%. Found: C, 87.21%; H, 7.65%; N, 4.74%; Mp: 136 °C; UV-vis in THF λ_max_ at 304, 318, 348, 364 and ~ 450 nm; PL in THF λ_max_ at 520 nm (excitation 450 nm).

*N-(4-((4-hexadecylphenylimino)methyl)benzylidene)pyren-1-amine*. (**AP1B**) Yield: 65%; ^1^H-NMR (ppm): δ= 8.88 (s, 1H, CH=N-Ar_pyrene_), 8.58 (s, 1H, CH=N-), 7.84-8.34 (m, 9H, H_Ar_, pyrene structure), 7.25-7.30 (m, 4H, 4 × H_Ar_), 7.04-7.08 (m, 4H, 4 × H_Ar_), 2.63-2.72 (m, 2H, CH_2_-Ar), 1.64 (m, 2H, Ar-CH_2_-CH_2_-), 1.30 (m, 26H, (CH_2_)_13_-CH_3_), 0.92-0.93 (m, 3H, CH_3_); Anal. Calcd. for C_46_H_52_N_2_ (632.92): C, 87.29%; H, 8.28%; N, 4.43%. Found: C, 87.21%; H, 8.45%; N, 4.37%; Mp: 99 °C. 

## 4. Conclusions

In summary, we have reported the synthesis and characterization of new symmetrical and unsymmetrical series of the calamitic azomethines with aromatic central core based on 2,5- thiophenedicarboxaldehyde and benzene-1,4-dicarboxaldehyde. The one group of the symmetrical compounds (i.e. the three benzene-1,4-dicarboxaldehyde derivatives) consisted of molecules containing two liquid crystal units with different length of the outer spacers, i.e. the length of the outer spacer is varied from 5 to 16 methylene groups. The second symmetrical group (i.e. the two 2,5- thiophenedicarboxaldehyde derivatives) consisted of molecules containing also two liquid crystal units with the 16 methylene or 16 methoxy groups as an outer spacers. The third group consist of unsymmetrical molecules containing three LC units with different length of the outer spacers (12 or 16 methylene groups). At an ambient temperature all of the azomethines were crystalline substances which melting point ranged from 92 °C to 282 °C. The mesomorphic properties of the compounds depend on both the length of the outer flexible spacers, the connection of the azomethine group with the rod, i.e. via phenyl or thiophene ring and symmetry of the molecule. The symmetrical compounds exhibited smectic and/or nematic phases whereas the unsymmetrical azomethines displayed rich polymorphism. 

The obtained unsymmetrical and symmetrical azomethines exhibit mesophases over a broad temperature range and can be used potentially in optoelectronics. For example, such LC compounds could be applied in organic light-emitting devices, whose operating temperatures could be higher than room temperature. Additionally, the obtained azomethines could be used in mixtures with other LCs for LC displays.
